# Engineering the Enantioselectivity of Yeast Old Yellow Enzyme OYE2y in Asymmetric Reduction of (*E*/*Z*)-Citral to (*R*)-Citronellal

**DOI:** 10.3390/molecules24061057

**Published:** 2019-03-18

**Authors:** Xiangxian Ying, Shihua Yu, Meijuan Huang, Ran Wei, Shumin Meng, Feng Cheng, Meilan Yu, Meirong Ying, Man Zhao, Zhao Wang

**Affiliations:** 1 Key Laboratory of Bioorganic Synthesis of Zhejiang Province, College of Biotechnology and Bioengineering, Zhejiang University of Technology, Hangzhou 310014, China; yushihuafx@163.com (S.Y.); meyroline.huang@gmail.com (M.H.); weiranzjut@163.com (R.W.); mengshuminzjut@163.com (S.M.); fengcheng@zjut.edu.cn (F.C.); mzhao@zjut.edu.cn (M.Z.); hzwangzhao@163.com (Z.W.); 2 College of Life Sciences, Zhejiang Sci-Tech Univeristy, Hangzhou 310018, China; meilanyu@zstu.edu.cn; 3 Grain and Oil Products Quality Inspection Center of Zhejiang Province, Hangzhou 310012, China; hz85672100@163.com

**Keywords:** asymmetric reduction, citral, citronellal, enantioselectivity, Old Yellow Enzyme, site-saturation mutagenesis, substrate binding mode

## Abstract

The members of the Old Yellow Enzyme (OYE) family are capable of catalyzing the asymmetric reduction of (*E*/*Z*)-citral to (*R*)-citronellal—a key intermediate in the synthesis of L-menthol. The applications of OYE-mediated biotransformation are usually hampered by its insufficient enantioselectivity and low activity. Here, the (*R*)-enantioselectivity of Old Yellow Enzyme from *Saccharomyces cerevisiae* CICC1060 (OYE2y) was enhanced through protein engineering. The single mutations of OYE2y revealed that the sites R330 and P76 could act as the enantioselectivity switch of OYE2y. Site-saturation mutagenesis was conducted to generate all possible replacements for the sites R330 and P76, yielding 17 and five variants with improved (*R*)-enantioselectivity in the (*E*/*Z*)-citral reduction, respectively. Among them, the variants R330H and P76C partly reversed the neral derived enantioselectivity from 32.66% *e.e.* (*S*) to 71.92% *e.e.* (*R*) and 37.50% *e.e.* (*R*), respectively. The docking analysis of OYE2y and its variants revealed that the substitutions R330H and P76C enabled neral to bind with a flipped orientation in the active site and thus reverse the enantioselectivity. Remarkably, the double substitutions of R330H/P76M, P76G/R330H, or P76S/R330H further improved (*R*)-enantioselectivity to >99% *e.e.* in the reduction of (*E*)-citral or (*E*/*Z*)-citral. The results demonstrated that it was feasible to alter the enantioselectivity of OYEs through engineering key residue distant from active sites, e.g., R330 in OYE2y.

## 1. Introduction

(*R*)-citronellal is a valuable intermediate for the synthesis of L-menthol through an acidic ene-cyclization and subsequent hydrogenation [1,2,3]. The potential of (*R*)-citronellal was also explored for the synthesis of natural vitamin E—a kind of fat-soluble vitamin with relatively high antioxidant ability [4,5]. The commercial Takasago process of (*R*)-citronellal began with myrcene to form an allylic amine, which underwent asymmetric isomerization in the presence of a 2,2′-bis(diphenylphosphino)-1,1′-binaphthyl(BINAP)-Rh complex and subsequent hydrolysis with acid to give enantiomerically pure (*R*)-citronellal [6]. In contrast to the three-step asymmetric synthesis from myrcene, the one-step enantioselective reduction of natural citral (the crude mixture of 60% geranial and 40% neral) was a simplified process for the synthesis of (*R*)-citronellal [7]. The enantioselective hydrogenation of (*E*/*Z*)-citral to afford an identical enantiomer remained challenging since the reduction of the geometric isomers geranial and neral by the same catalyst usually yielded the enantiocomplementary products. In organocatalysis, the enantioselective hydrogenation of (*E*/*Z*)-citral to yield (*R*)-citronellal required the use of a dual catalyst system comprising of Pd/BaSO4 and chiral 2-diarylmethylpyrrolidine [8]. However, the obtained (*R*)-citronellal with 89% *e.e.* was insufficient for broad industrial applications.

To develop a greener and cost-effective alternative to organocatalysis, Old Yellow Enzymes (OYEs; EC 1.6.99.1) as biocatalysts have been widely investigated, which are capable of catalyzing the C=C bond reduction of α,β-unsaturated compounds such as (*E*/*Z*)-citral [9,10,11,12,13]. Past efforts have been made on the discovery of new, improved biocatalysts for suitable enantioselectivity and activity. Bacterial OYEs commonly produced (*S*)-citronellal from (*E*/*Z*)-citral reduction, while the counterparts from yeasts mainly afforded to (*R*)-enantiomer [10]. Representative yeast OYEs have been well characterized, including OYE2.6 from *Pichia stipites* [9], OYE1 from *Saccharomyces pastorianus*, and OYE2 and OYE3 from *Saccharomyces cerevisiae* [14,15]. So far, the application of OYE-mediated citral reduction still suffers from insufficient enantioselectivity and activity. Protein engineering has emerged as an attractive and powerful strategy for improving enzyme activity and selectivity [16,17,18,19]. The circular permutation of OYE1 from *S. pastorianus* yielded the variants exhibiting over an order of magnitude improved catalytic activity [20]. The activity improvement in the protein engineering of yeast OYEs commonly varied by substrate. The variant P295A of OYE1 from *S. pastorianus* showed three- and seven-fold activity for (*R*)- and (*S*)-carvone higher than those of wild type enzyme, respectively; however, it had no significant improvement for geranial and neral [21].

With regard to the alteration of OYE enantioselectivity, one of representative examples was the variant W116F of OYE1 from *S. pastorianus* partly reversed the enantioselectivity in the neral reduction from 19% *e.e.* (*S*) to 65% *e.e.* (*R*) as compared to the wild type [22]. In contrast to the substrate binding mode of the wild type enzyme, the W116F mutation enabled the substrate to bind with a flipped orientation in the active site, and thus reverse the enantioselectivity, while maintaining the same mechanism of trans-hydrogenation of C=C bond [23,24]. W116 is not the sole determinant of enantioselectivity in OYEs, and the enantioselectivity switches seemed to vary by enzyme: Y78, I113, and F247 in OYE2.6 [25]; C26, I69, and H167 in ene reductases YqjM [26]; and W66 and W100 in OYE from *Gluconobacter oxydans* (Gox0502) [27]. The study of enantioselectivity alteration in OYEs rarely use citral as substrate. The latest example was the NADH-dependent cyclohexenone ene reductase from *Zymomonas mobilis* (NCR), in which W66 was critical for controlling the orientation of (*E*/*Z*)-citral binding and, thus, the variant W66A/I231A of NCR reversed the geranial derived enantioselectivity from 99% *e.e.* (*S*) to 63% *e.e.* (*R*) [28].

**Scheme 1:**
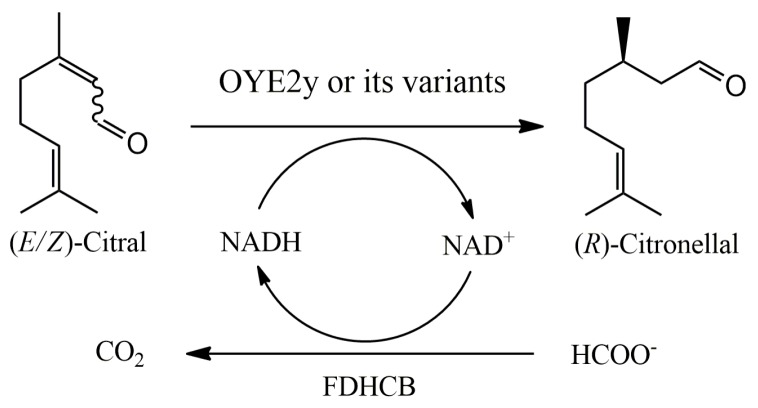
OYE-mediated asymmetric reduction of (*E*/*Z*)-citral to (*R*)-citronellal coupled with formate dehydrogenase from *Candida boidinii* (FDHCB)-catalyzed NADH regeneration. The reactions were conducted at 37 °C and 200 rpm for 11 h.

The study aims to develop the asymmetric reduction of (*E*/*Z*)-citral to (*R*)-citronellal using engineered OYE coupled with formate dehydrogenase for NADH regeneration (Scheme 1). The Old Yellow Enzyme from *S. cerevisiae* CICC1060 (OYE2y) was cloned, overexpressed, and purified, which reduced geranial and neral to citronellal with 82.87% *e.e.* (*R*) and 32.66% *e.e.* (*S*), respectively. OYE2y was chosen for enantioselectivity alteration since the wild type enzyme showed higher enantioselectivity than OYE1 from *S. pastorianus* and OYE2 and OYE3 from *S. cerevisiae* in the (*E*/*Z*)-citral reduction [29]. The key residues for the enantioselectivity of OYE2y were identified through the combination of sequence alignment and single-point mutations. Relying on subsequent site-saturation mutagenesis, the OYE2y variants with double substitutions exhibited full (*R*)-enantioselectivity in the reduction of (*E*)-citral or (*E*/*Z*)-citral. In addition, the role of key residues and the substrate binding modes were examined via homology modeling and molecular docking.

## 2. Results

### 2.1. OYE2y-Mediated Reduction of (E/Z)-Citral

**Figure 1:**
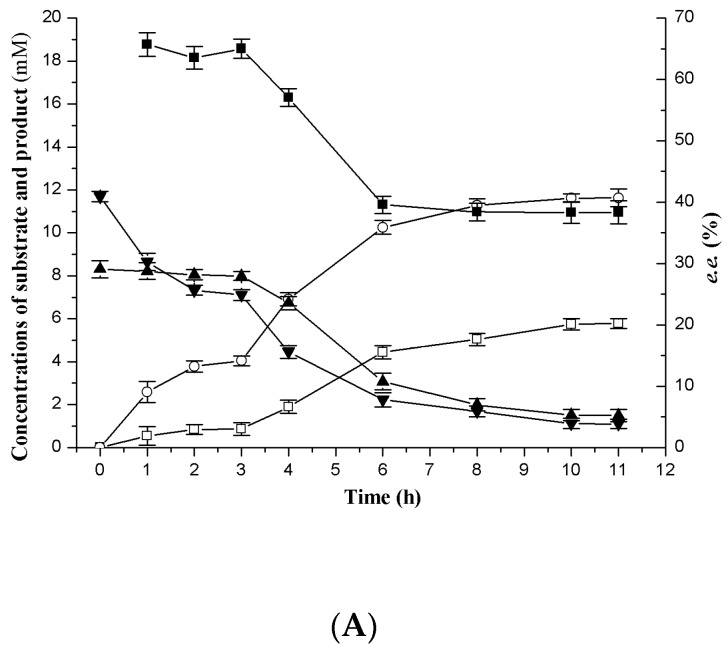

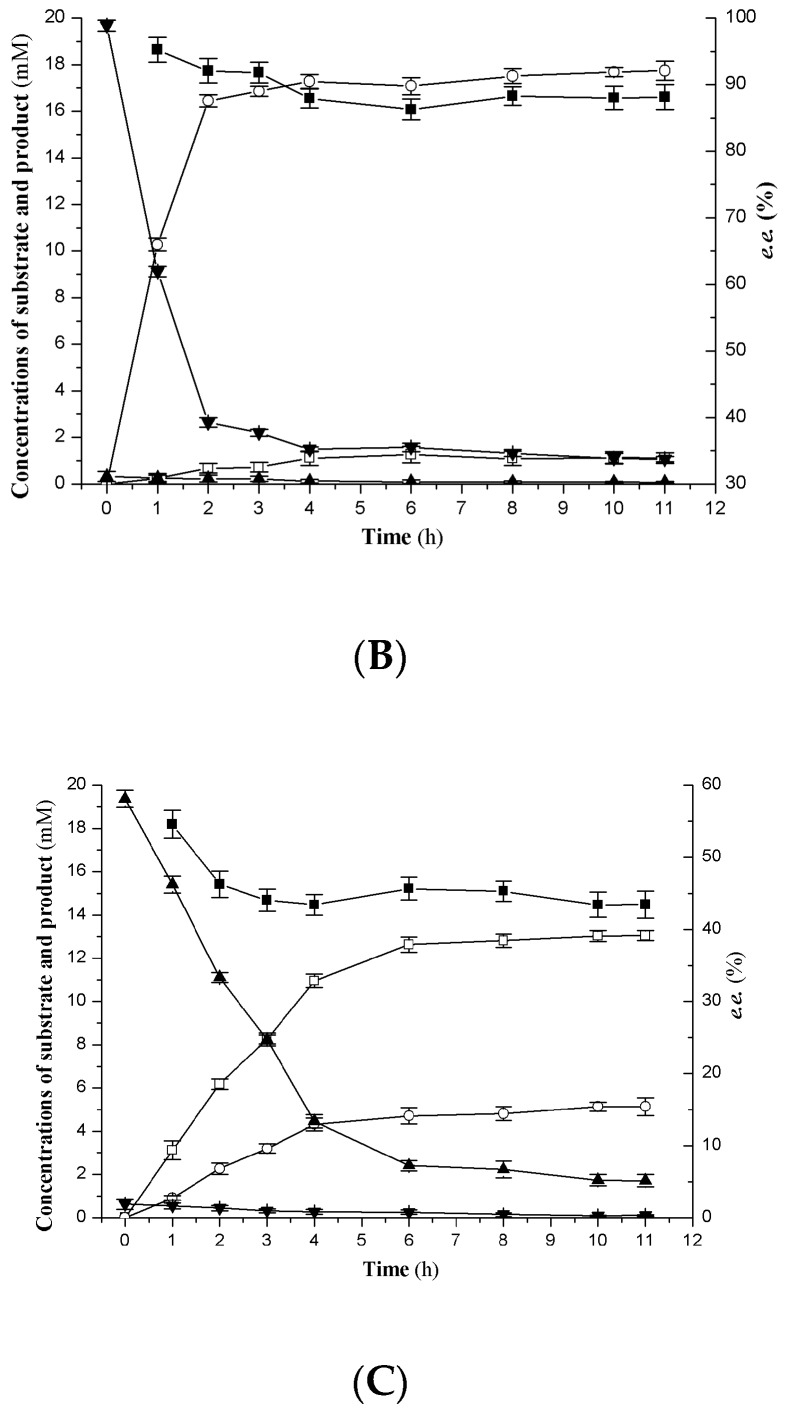
Asymmetric reduction of (*E*/*Z*)-citral (**A**), (*E*)-citral (**B**), and (*Z*)-citral (**C**) using the purified OYE2y. □, (*R*)-citronellal; ○, (*S*)-citronellal; ▲, geranial; ▼, neral; ■, the *e.e.* value of hydrogenated product. Data present mean values ± SD from three independent experiments.

The yeast Old Yellow Enzyme OYE2y was heterologously expressed in *Echerichia coli* BL21(DE3), and the resulting recombinant OYE2y with *N*-terminal His tag was purified using affinity chromatography. The enzyme OYE2y with 400 amino acid residues shared the sequence identities of 91.50%, 98.75%, and 81.25% to OYE1 from *S. pastorianus* and OYE2 and OYE3 from *S. cerevisiae* [14], respectively. To investigate the enantioselectivity of OYE2y in citral reduction, the purified OYE2y rather than the whole-cell biocatalyst was used to avoid side reactions. OYE2y accepted NADH or NADPH as coenzyme, and formate/formate dehydrogenase system was used for NADH regeneration in this study. The composition of (*E*/*Z*)-citral was determined to contain 58.45% geranial and 41.55% neral. During the first 3 h, the concentration of geranial decreased significantly faster than that of neral. Meanwhile, the concentration of (*R*)-citronellal increased rapidly, and the *e.e.* value was kept at a higher level of >60% (*R*) (Figure 1A). Then, the conversion rate of neral turned faster from 3 h to 5 h, resulting in the decreasing of *e.e.* value from 65.02% (*R*) to 40.26% (*R*). After 6 h, the conversion rate of neral was nearly parallel to that of geranial, meanwhile the *e.e.* values were kept almost constant. The 11 h reaction was completed with 89.51% yield, giving (*R*)-citronellal with an *e.e.* value of 38.13% (*R*). The time course of (*E*/*Z*)-citral reduction clearly indicated that the ratio of geranial and neral significantly affected the *e.e.* value of (*R*)-citronellal, which was consistent with previous observations [7,14]. In addition, the isomerization of geranial and neral occurred under some conditions [9,30]. Thus, the use of freshly prepared geranial and neral with high purity was necessary to study the enantioselectivity of OYEs.

Considering the high cost of commercial products, geranial and neral with high purity were prepared according to the previous procedure with improvements [31]. Based on the optimized conditions, the yields of (*E*)-citral and (*Z*)-citral were increased up to 99.39% and 99.35%, respectively. The obtained (*E*)-citral sample contained 98.38% geranial and 1.62% neral, and the obtained (*Z*)-citral sample contained 96.84% neral and 3.16% geranial. When the enzyme OYE2y was tested with newly prepared (*E*)-citral or (*Z*)-citral for 4 h, the enantioselectivity of OYE2y stayed at a relatively constant level (Figure 1B,C). The (*E*)-citral and (*Z*)-citral-derived *e.e.* values after 11 h reduction were 82.87% (*R*) and 32.66% (*S*), respectively.

### 2.2. Identification of Key Residues for the Enantioselectivity of OYE2y

**Figure 2:**
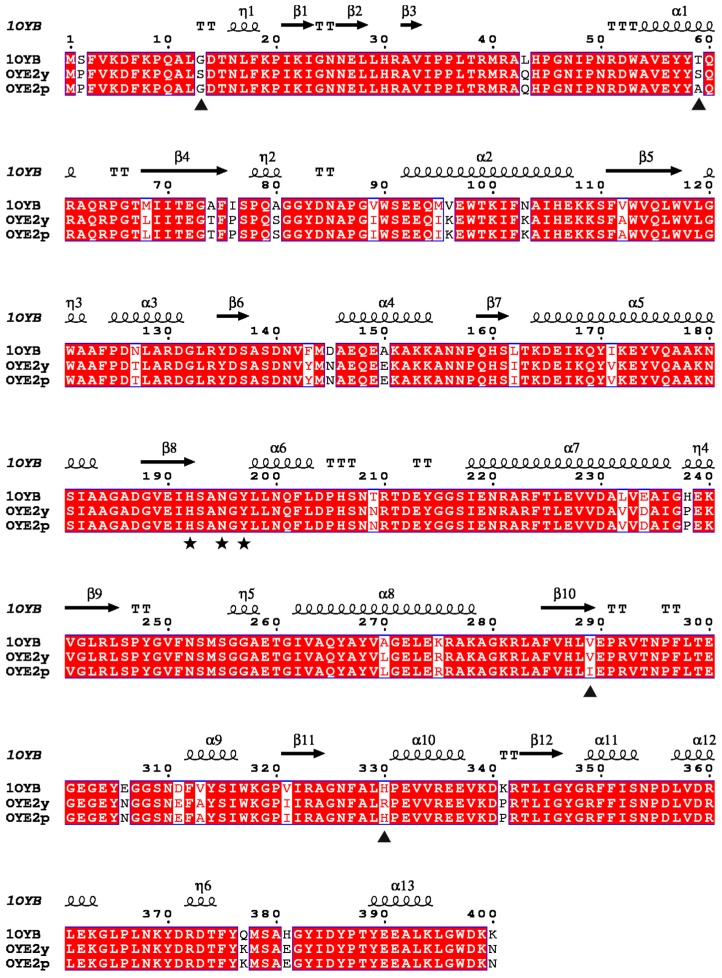
Structure-related sequence alignment between OYE2y and its homologous OYEs. 1OYB: PDB code of OYE1 from *S. pastorianus*; OYE2p: NAD(P)H-dependent enoate reductase from *S. cerevisiae* YJM1341. The secondary structural elements of 1OYB (α-helices, β-strands, T-turns, and η-helices) were indicated above the aligned sequences. The numbering shown was from 1OYB. A red background highlights conserved residues. ▲, the positions where the amino acid residues differed between OYE2y and OYE2p; ★, key residues for catalytic activity. The figure was produced using ESPript 3.0 [32].

It was previously reported that the variant W116F of OYE1 from *S. pastorianus* partly reversed the enantioselectivity in the neral reduction [22]. However, the same substitution at site W117 corresponding to W116 in OYE1 even lowered the *e.e.* value from 38.13% (*R*) to 24.01% (*R*) when (*E*/*Z*)-citral was tested as substrate, indicating that the enantioselectivity switch for OYE1 and OYE2y seemed different. Furthermore, the NAD(P)H-dependent enoate reductase (OYE2p) from *S. cerevisiae* YJM1341 was newly discovered for asymmetric reduction of (*E*/*Z*)-citral to (*R*)-citronellal with the *e.e.* value of 88.8% (*R*), with four amino acid residues—G13, A59, I289, and H330—in OYE2p different from S13, S59, V289, and R330 in OYE2y (Figure 2). Considering the difference of enantioselectivity between OYE2p and OYE2y, it was expected that S13, S59, V289, and/or R330 might be critical for the enantioselectivity. Then, the single substitutions were conducted to evaluate this expectation. The catalytic performance of the variants S13G, S59A, and V289I was similar to that of OYE2y (Table 1). The substitution R330 to H significantly increased the (*R*)-enantioselectivity from 38.13% to 86.88% when (*E*/*Z*)-citral was used as substrate. Remarkably, the enantioselectivity was reversed from 32.66% (*S*) to 71.92% (*R*) when (*Z*)-citral was tested. Except for sequence alignment, the identification of key residues was conducted through the single mutation on the randomly-selected residues. Through multiple mutation attempts, the variant P76M was discovered to benefit the (*R*)-enantioselectivity of OYE2y in the citral reduction (Table 1). The substitution P76 to M increased the (*E*/*Z*) citral-derived *e.e.* value from 38.13% (*R*) to 57.60% (*R*), while the enantioselectivity in the reduction of (*Z*)-citral was lowered from 32.66% (*S*) to 4.76% (*S*). Thus, both R330 and P76 were chosen as the targets for subsequent site saturation mutagenesis.

### 2.3. Site-Saturation Mutagenesis of R330 in OYE2y

All R330X variants of OYE2y (X = one of the other 19 amino acids) were successfully expressed in *E. coli*. After the cells were harvested by centrifugation and then disrupted by ultrasonication, each variant with *N*-terminal His tag was purified using affinity chromatography. As shown in Figure S1, all 19 variant proteins remained in the soluble fraction, revealing that these substitutions did not decrease the solubility. In comparison with the wild type OYE2y, the R330 variants of OYE2y fell into three categories: R330P without catalytic activity, R330Y with similar (*R*)-stereoselectivity to OYE2y, and the other 17 variants with improved (*R*)-stereoselectivity (Table 2). R330P did not retain the yellow color, suggesting that its substitution might deactivate the coenzyme binding. When (*E*/*Z*)-citral was tested as substrate, the variants except R330Y and R330P increased the (*R*)-stereoselectivity but decreased the product yield to some extent. In contrast to the reduction of (*E*)-citral, those 17 variants showed more significant improvement of (*R*)-enantioselectivity in the reduction of (*Z*)-citral. Among them, the variants R330H, R330D, and R330W had superior catalytic performance in terms of activity and enantioselectivity.

### 2.4. Site-Saturation Mutagenesis of P76 in OYE2y

Similar to R330X variants, all P76X variants of OYE2y were successfully expressed in *E. coli* and purified (Figure S2). However, the number of the P76 variants in the category without catalytic activity (P76Y, P76Q, P76D, P76E, P76R, P76H, P76F, P76W, and P76K) was obviously greater than that of R330 variants, suggesting that P76 could be also critical for the activity. The category with significantly improved enantioselectivity included P76C, P76S, P76M, P76G, and P76N, whereas the other five variants (P76A, P76V, P76T, P76L, and P76I) showed similar catalytic performance to that of OYE2y (Table 3). Similar to the trend in the R330X variants, higher (*R*)-stereoselectivity of OYE2y variants was accompanied by lower product yields. When (*E*/*Z*)-citral was used as substrate, the substitution of P76 to C increased the *e.e.* value from 44.13% (*R*) to 69.92% (*R*), but the yield was lowered from 89.51% to 49.65%. Particularly, the *e.e.* value in the reduction of (*Z*)-citral was partly reversed from 32.66% (*S*) to 37.50% (*R*).

### 2.5. Evaluation of Double Substitution at Sites P76 and R330 of OYE2y

To investigate the effect of the double substitutions on P76 and R330, we firstly conducted site-directed mutagenesis of P76 to C and R330 to H, D, W, and C, resulting in the four variants (Table 4). The *e.e.* values and yields of the resulting variants were higher than those of the variant P76C, but similar to those of the corresponding R330H, R330D, R330W, and R330C. On the other hand, the other set of double substitutions was created by site-directed mutagenesis of R330 to H and P76 to M, G, and S (Table 4). Among them, the (*E*)-citral-derived *e.e.* values were significantly increased up to >99% (*R*) with relatively higher yields (64.09%~73.88%). In the (*E*/*Z*)-citral reduction, the variants P76M/R330H, P76M/R330H, and P76M/R330H also exhibited full (*R*)-enantioselectivity despite lower product yields (9.12–15.83%).

## 3. Discussion

For the synthesis of (*R*)-citronellal from OYE-mediated citral reduction, low-cost (*E*/*Z*)-citral is the industrial desire in contrast to geranial and neral [9]. However, (*E*/*Z*)-citral reduction remains challenging due to limited chemoselectivity and enantioselectivity. On the one hand, the presence of multiple C=C and C=O double bonds of citral makes the whole-cell biocatalyst impossible to avoid side reactions [33]. Thus, the purified enzyme is commonly required as biocatalyst. On the other hand, the hydrogenated products from the geometric isomers geranial and neral are usually enantiocomplementary when the wild type OYE was used as biocatalyst, reducing the reaction’s enantioselectivity. The features of (*E*/*Z*)-citral reduction make it difficult to implement a high-throughput screening (HTS) method for determining the enantioselectivity of the large variant libraries [34,35,36]. Typically, the samples must be examined individually by chiral GC, which requires at least thirty minutes per sample. To keep the variant library as minimum as possible, the best strategy for enantioselectivity alteration turns to be site-saturation mutagenesis of individual key residue(s) rather than the HTS-based directed evolution [18,37,38]. Based on the strategy of site-saturated mutagenesis, several groups have successfully increased (*R*)-enantioselectivity in the (*E*/*Z*)-citral reduction, and our study further demonstrated that it was feasible to achieve the full (*R*)-enantioselectivity in the OYE-mediated (*E*/*Z*)-citral reduction through protein engineering.

The R330X and P76X variant libraries yielded 17 and five variants with improved (*R*)-enantioselectivity, respectively, indicating that the subtle change of structure would significantly affect the enantioselectivity. In the variants R330H and P76C, the amino acid pair R and H possessed electrically charged side group, while the side groups of the amino acid pair P and C was polar and uncharged. It was suggested that the substitution could give priority to amino acid residue(s) with similar side group in terms size, polarity and charge, if no clear structure–function relationship was available. Furthermore, the (*Z*)-citral-derived *e.e.* value of R330H (71.92%, *R*) was much higher than that of OYE2p (26.5%, *R*) [7], suggesting that the role of S13, S59, and V289 could not be ignored for the (*R*)-enantioselectivity. For the single substitutions, the (*R*)-enantioselectivity improvement in the (*E*/*Z*)-citral reduction was mainly attributed to the enantioselectivity inversion in the (*Z*)-citral reduction, meanwhile, the same variant reduced (*E*)-citral to (*R*)-citronellal with similar *e.e.* values. Different from the single substitutions, the double substitutions led to further improved (*R*)-stereoselectivity in the (*E*)-citral reduction rather than (*Z*)-reduction, which was rarely observed in the enantioselectivity alternation of OYEs.

**Figure 3:**
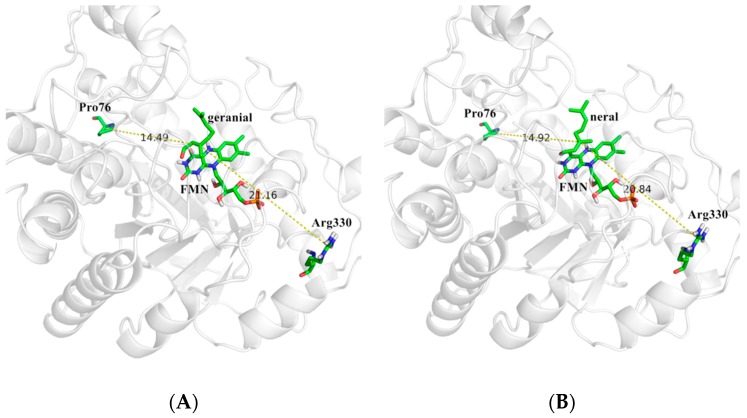
The residues targeted for site-saturation mutagenesis in the homology model of OYE2y. The model structure of OYE2y was constructed with the crystal structure of OYE1 (PDB code: 1OYB) as template. Distances between the C_β_ atom of geranial (**A**) and neral (**B**) and side chains of P76 and R330 were determined. Green, carbon atom; blue, nitrogen atom; tangerine, oxygen atom; white, hydrogen atom; orange, phosphorus atom.

**Figure 4:**
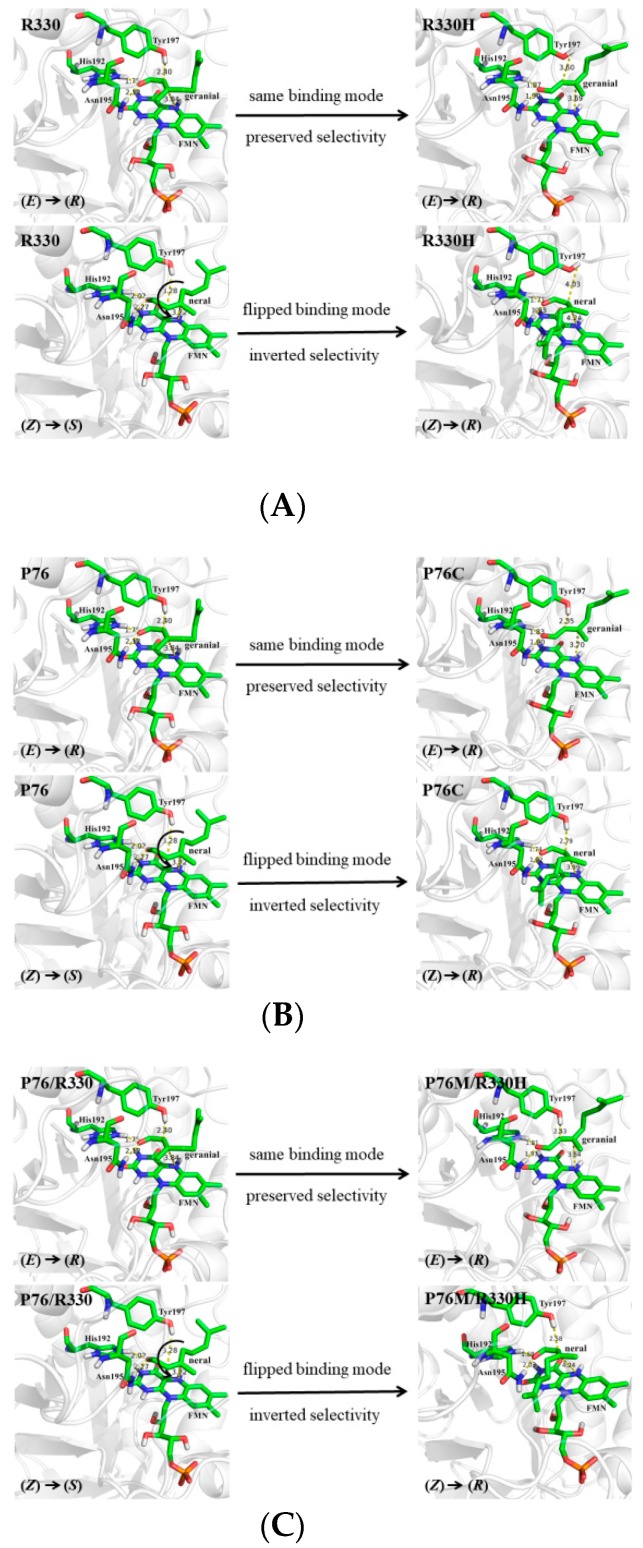
The binding modes of citral isomers in OYE2y and its variants R330H (**A**), P76C (**B**), and P76M/R330H (**C**) leading to either (*R*)- or (*S*)-citronellal. The catalytic residues H192, N195, Y197, and the prosthetic group FMN were depicted. Green, carbon atom; blue, nitrogen atom; tangerine, oxygen atom; white, hydrogen atom; orange, phosphorus atom.

To get detailed insights into the molecular mechanism, the models of OYE2y were created from the OYE1 structure (PDB code: 1OYB) using SWISS-MODEL and molecular docking was conducted using the program AutoDock Vina. The substrate and FMN were docked in silico into the models of OYE2y and its variants. In the model of wild type OYE2y, a conserved H192/N195 pair formed hydrogen bonds with the carbonyl oxygen of α,β-unsaturated carbonyl compounds; a hydride was enantioselectively transferred to the substrate C_β_ atom from FMNH_2_; and the Y197 residue provided a proton to the substrate C_α_ atom as an electron acceptor [17]. From the model of wild type OYE2y, the distances from C_β_ of neral to the side groups of P76 and R330 were calculated to be 14.92 and 20.84 Å, respectively (Figure 3). Similarly, the distances from C_β_ of geranial to the side groups of P76 and R330 were calculated to be 14.49 and 21.16 Å, respectively. It was assumed that the residues affecting the enantioselectivity of OYEs might directly interact with the substrate [25]. Our results indicated that the residue distant from active sites, e.g., R330 in OYE2y, could also be pivotal for determining the enantioselectivity of OYEs. Furthermore, substrate modeling into the wild type enzyme revealed two different binding modes for the two isomers geranial and neral, leading to products with different enantioselectivity. The docking analyses of variants P76C, R330H, and P76M/R330 suggested that the reversed enantioselectivity in the neral reduction was due to the flipped binding orientation that placed the opposite face of the alkene above the *si* face of the FMN cofactor [24]. Meanwhile, the same variant reduced (*E*)-citral with preserved (*R*)-enantioselectivity derived from the same binding orientation as wild type OYE2y (Figure 4). Although it was acceptable that enantioselectivity was controlled by tuning the orientation of substrate in the binding sites, how subtle changes control the orientation of substrate binding remains to be unraveled, and X-ray crystallography of the variants in the future study would benefit to clarify the subtle structural differences at the substrate-binding site.

## 4. Materials and Methods

### 4.1. Organisms and Chemicals

The organism *S. cerevisiae* CICC1060 was purchased from the China Center of Industrial Culture Collection (CICC, Beijing, China). *S. cerevisiae* CICC1060 was cultured with YPD medium (tryptone 20 g/L, yeast extract 10 g/L, and glucose 20 g/L) at 30 °C for 24 h. The pEASY-E1 expression vector from TransGen Biotech Co., Ltd (Beijing, China) was used for overexpression of the enzyme OYE2y, and the *E. coli* strain BL21(DE3) was used as the host. *E. coli* cultures were grown routinely in Luria Bertani (LB) medium at 37 °C for 12 h.

The standards (*S*)-citronellal, (*R*)-citronellal, and (*S*/*R*)-citronellal were obtained from Sigma-Aldrich (Shanghai) Trading Co., Ltd. (Shanghai, China). Other chemicals of analytical grade were purchased from Sangon Biotech Co. Ltd (Shanghai, China) or Shanghai Jingchun Reagent Co., Ltd (Shanghai, China). The site-directed mutagenesis kit and the restriction enzyme *Dpn* I were obtained from Vazyme Biotech Co., Ltd. (Nanjing, China). KOD DNA polymerase was purchased from TransGen Biotech Co., Ltd (Beijing, China). The Ni-NTA-HP resin column for protein purification was obtained from GE Healthcare Life Sciences (Shanghai, China).

### 4.2. Preparation of (Z)-Citral and (E)-Citral

(*Z*)-citral and (*E*)-citral were prepared by a modification of the procedure described previously [38]. Activated MnO_2_ (1.15 g) was added into a 50-mL three-necked bottom round flask which atmosphere was replaced with N_2_. One-hundred milligrams of geraniol or nerol was dissolved in 16 mL dry hexane and was charged in the flask to initiate the alcohol oxidation. The reaction was maintained at 0 °C and 450 rpm for 6 h. Then, the reaction solution was filtered through a filter paper and hexane in the filtrate was removed by vacuum evaporation at 45 °C. Finally, an aliquot of the collected product (*E*)-citral or (*Z*)-citral was dissolved in ethyl acetate and subjected to the analyses of gas chromatography (GC) and gas chromatography–mass spectrometry (GC–MS).

### 4.3. Cloning, Expression, and Purification of OYE2y

The gene encoding OYE2y was PCR-amplified from the genomic DNA of *S. cerevisiae* CICC1060 using a set of primers: Forward, 5′-ATGCCATTTGTTAAGGACTTTAAGCCAC-3′; Reverse, 5′-TTAATTTTTGTCCCAACCGAGTTTTAGAGC-3′. The conditions for PCR amplification of the *oye2y* gene were 94 °C for 2 min for initial denaturalization, 30 cycles of 94 °C for 30 s, 57 °C for 30 s, 72 °C for 80 s, and 72 °C for 10 min for the final extension.

Following the procedure of expression and purification of ReBDH [39], the PCR products were purified and then ligated with the expression vector pEASY-E1 through the AT ligation strategy. The recombinant plasmid harboring the *oye2y* gene, designated as pEASY-E1-*oye2y*, was verified by DNA sequencing (Sangon Biotech, Shanghai, China) and then transformed into *E. coli* BL21 (DE3) competent cells, resulting in the recombinant strain *E. coli* BL21(DE3)/pEASY-E1-*oye2y*. The recombinant cells containing pEASY-E1-*oye2y* were grown in the LB medium with 100 μg/mL ampicillin at 37 °C and 200 rpm until the OD_600_ was 0.6–0.8, and then 0.2 mM isopropyl β-d-1-thiogalactopyranoside (IPTG) was supplemented to initiate the induction at 23 °C and 160 rpm. After 12 h of growth, recombinant *E. coli* cells were harvested by centrifugation and further washed using 50 mM Tris-HCl buffer (pH 8.0). The cells were disrupted through ultrasonication for 10 min, and the cell debris and cell lysate were removed by centrifugation to result in a clear cell extract. The crude cell extracts containing OYE2y was applied to a Ni-NTA chelating affinity column equilibrated with the binding buffer (5 mM imidazole and 300 mM NaCl dissolved in 50 mM Tris-HCl, pH 8.0). Unbound proteins were washed off by the application of the binding buffer. The recombinant OYE2y was eluted with 100 mM imidazole in 50 mM Tris-HCl (pH 8.0), desalted with 50 mM Tris-HCl buffer (pH 8.0) by ultrafiltration and then stored at −20 °C for further study.

### 4.4. Construction of OYE2y Variants by Site-Directed Mutagenesis

The variants with single or double substitutions were constructed by site-directed mutagenesis according to the QuikChange Mutagenesis Kit. PCR amplification to introduce substitution was performed in 50 μL of standard PCR mixture with 50 ng of template plasmid DNA and 15 pmol each of the appropriate set of primers using the following temperature cycle; 30 s at 95 °C, followed by 30 cycles of 95 °C for 15 s, appropriate annealing temperature (55~61 °C) for 15 s, and 72 °C for 80 s, and the final extension of 5 min at 72 °C. The plasmid pEASY-E1-*oye2y* was used as template DNA in the single substitution, while the creation of double mutants was based on the generated OYE2y P76C or R330H gene as the template. The primer sets for single or double substitutions were shown in Tables S1 and S2. The amplified PCR fragments were digested with the restriction enzyme *Dpn* I at 37 °C for 1 h, and then the digested DNA was directly introduced into *E. coli* strain BL21(DE3). Each constructed plasmid was confirmed by sequencing. Expression and purification of the resulting OYE2y variants were conducted using the same procedure as OYE2y.

### 4.5. Homology Modeling and Molecular Docking

Using the crystal structure of OYE1 from *S. pastorianus* (PDB number: 1OYB) as a template [40], the structural model of OYE2y was obtained by a homology modeling strategy [41,42]. Molecular docking simulation was performed by AutoDock Vina [43] when the binding package was set at a distance of 15 Å from FMN N_5_ atom. (*E*)-citral or (*Z*)-citral acted as a ligand to molecular docking with OYE2y, and the calculation of geometric parameters and ligand structure was performed by ChemBioDraw 12.0 (CambridgeSoft, Cambridge, MA, USA). To make the results more accurate, 100 consecutive runs were performed and the highest ranked score from each run was used to calculate the average score of each flexible ligand configuration. The optimal configuration and the resulting substrate–enzyme complexes were further processed using the PYMOL software [44]. The candidate complexes were acceptable when they met both criteria: (1) the substrate carbonyl oxygen should be capable of forming hydrogen bonds with the side chains of both H192 and N195 and (2) the distance between the FMN N_5_ atom and the β-unsaturated carbon of the substrate molecule was be in an appropriate range from 3.5 Å to 4.1 Å [22,45,46].

### 4.6. Asymmetric Reduction of Citral Mediated by OYE2y or Its Variants

The reaction mixture (1 mL) contained 50 mM PIPES buffer (pH 7.0), 20 mM substrate, 1 mg OYE2y or its variant, 0.6 U formate dehydrogease from *Candida boidinii* (FDHCB), and 100 mM sodium formate, 0.96 mM NAD^+^. The substrate included (*Z*)-citral, (*E*)-citral, or (*E*/*Z*)-citral, which stock solution was 200 mM substrate in isopropanol. The overexpression and purification of FDHCB were conducted according to the procedure described previously [39]. The reaction was conducted at 30 °C and 200 rpm for 11 h, unless otherwise specified. The reaction mixture was centrifuged to remove the cells, and the resulting supernatant was extracted with equal volume of ethyl acetate at 30 °C and 200 rpm for 2 h. Finally, the solvent phase was collected, dried over anhydrous sodium sulfate and subjected to the analyses of GC and GC–MS.

### 4.7. Analyses of GC and GC–MS

(*Z*)-citral, (*E*)-citral, (*S*)-citronellal, and (*R*)-citronellal were determined by GC (Agilent 6890N) equipped with an FID detector and chiral capillary BGB-174 column (BGB Analytik, Böckten, Switzerland, 30 m × 250 μm × 0.25 μm). The flow rate and split ratio of N_2_ as the carrier gas were set as 1.38 mL/min and 1:100, respectively. Both injector and detector were kept at 250 °C. The column temperature program was listed as follows; initial temperature of 90 °C for 25 min, 20 °C/min ramp to 150 °C for 3 min, and 30 °C/min ramp to 180 °C for 3 min. The injection volume was 1 μL. The retention times of (*S*)-citronellal, (*R*)-citronellal, (*Z*)-citral, and (*E*)-Citral were 22.459 min, 23.067 min, 29.164 min and 30.398 min, respectively (Figure S3).

(*S*)-citronellal, (*R*)-citronellal, (*Z*)-citral, and (*E*)-Citral were validated through GC–MS analysis (Figure S4). The GC–MS analysis (Agilent7890A/5975C, Agilent Technologies Inc., Santa Clara, CA, USA) comprised the following parameters; auxiliary heating zone temperature, 250 °C; MS quadrupole temperature, 150 °C; ion source temperature, 230 °C; scan quality range, 30–500 amu; emission current, 200 μA; and electron energy, 70 eV.

### 4.8. Nucleotide Sequence Accession Number

The gene encoding OYE2y has been deposited in the GenBank database under the accession numbers of MK372229.

## 5. Conclusions

In summary, significant increase of (*R*)-enantioselectivity in the (*E*/*Z*)-citral reduction was achieved by saturation mutagenesis of P76 and R330 in OYE2y. Remarkably, the variants P76M/R330H, P76G/R330H, and P76S/R330H exhibited full (*R*)-enantioselectivity in the reduction of (*E*)-citral or (*E*/*Z*)-citral. The variants with improved (*R*)-enantioselectivity usually came along with lower catalytic activities, indicating that the sites P76 and R330 were important for enantioselectivity as well as activity. In contrast to P76, R330 was relatively distant from active sites and its substitutions brought more beneficial impacts on enantioselectivity. Our results proved that it was reasonable to alter the enantioselectivity of OYE2y by saturation mutagenesis of key residue distant from active sites.

## Figures and Tables

**Table 1:** The catalytic performance of OYE2y and its variants S13G, S59A, P76M, V289I, and R330H ^a^.

Enzyme	(*E*)-Citral		(*Z*)-Citral		(*E*/*Z*)-Citral	
	*e.e.* (%)	Yield (%)	*e.e.* (%)	Yield (%)	*e.e.* (%)	Yield (%)
S13G	83.19 ± 1.57 (*R*)	95.12 ± 2.10	45.29 ± 1.46 (*S*)	94.60 ± 1.22	37.35 ± 1.63 (*R*)	96.34 ± 1.87
S59A	80.69 ± 2.21 (*R*)	90.32 ± 1.54	30.96 ± 1.25 (*S*)	90.88 ± 2.23	41.66 ± 1.59 (*R*)	93.48 ± 1.07
V289I	75.18 ± 1.70 (*R*)	94.88 ± 1.81	37.45 ± 1.55 (*S*)	91.71 ± 2.10	35.52 ± 2.42 (*R*)	90.78 ± 0.93
R330H	88.08 ± 1.39 (*R*)	71.23 ± 0.85	71.92 ± 1.34 (*R*)	64.12 ± 1.33	86.88 ± 1.36 (*R*)	52.83 ± 0.75
P76M	86.22 ± 0.75 (*R*)	80.39 ± 0.64	4.76 ± 1.50 (*S*)	55.19 ± 0.95	57.60 ± 0.92 (*R*)	61.53 ± 0.89
OYE2y	82.87 ± 0.98 (*R*)	92.20 ± 1.07	32.66 ± 1.77 (*S*)	88.65 ± 1.49	38.13 ± 1.55 (*R*)	89.51 ± 1.68

^a^ Data present mean values ± SD from three independent experiments. (*E*)-citral contained 98.38% geranial and 1.62% neral, (*Z*)-citral contained 96.84% neral and 3.16% geranial, and (*E*/*Z*)-citral contained 58.45% geranial and 41.55% neral.

**Table 2:** The catalytic performance of OYE2y and its R330X variants ^a^.

Enzyme	(*E*)-Citral		(*Z*)-Citral		(*E*/*Z*)-Citral	
	*e.e.* (%)	Yield (%)	*e.e.* (%)	Yield (%)	*e.e.* (%)	Yield (%)
R330H	88.08 ± 1.39 (*R*)	71.23 ± 0.85	71.92 ± 1.34 (*R*)	64.12 ± 1.33	86.88 ± 1.36 (*R*)	52.83 ± 0.75
R330D	92.42 ± 2.30 (*R*)	63.12 ± 1.93	73.56 ± 0.51 (*R*)	36.41 ± 1.94	80.30 ± 1.85 (*R*)	62.70 ± 0.85
R330W	95.05 ± 1.85 (*R*)	47.32 ± 1.11	72.19 ± 1.09 (*R*)	53.38 ± 1.12	79.71 ± 1.03 (*R*)	70.98 ± 1.15
R330I	87.38 ± 1.11 (*R*)	31.45 ± 0.40	20.93 ± 1.21 (*R*)	13.19 ± 0.23	74.52 ± 0.69 (*R*)	28.17 ± 0.68
R330L	89.64 ± 0.79 (*R*)	75.14 ± 1.25	45.59 ± 0.75 (*R*)	55.72 ± 0.59	72.58 ± 1.32 (*R*)	67.45 ± 1.21
R330F	89.40 ± 1.05 (*R*)	89.86 ± 1.74	57.68 ± 0.86 (*R*)	78.39 ± 1.34	72.50 ± 1.46 (*R*)	80.49 ± 1.69
R330E	89.22 ± 0.61 (*R*)	80.92 ± 0.80	47.80 ± 1.14 (*R*)	47.82 ± 1.11	69.42 ± 2.34 (*R*)	72.46 ± 1.02
R330A	88.10 ± 1.37 (*R*)	83.10 ± 2.11	51.07 ± 1.23 (*R*)	49.12 ± 1.63	69.42 ± 1.11 (*R*)	69.61 ± 0.72
R330T	85.74 ± 2.33 (*R*)	83.33 ± 2.06	27.97 ± 0.48 (*R*)	43.83 ± 0.87	69.12 ± 2.32 (*R*)	46.61 ± 0.29
R330N	92.15 ± 2.49 (*R*)	90.55 ± 1.85	41.87 ± 0.73 (*R*)	72.44 ± 1.61	68.19 ± 1.56 (*R*)	78.89 ± 1.98
R330V	89.66 ± 1.50 (*R*)	91.10 ± 1.01	24.39 ± 1.55 (*R*)	65.15 ± 1.69	67.68 ± 1.42 (*R*)	75.77 ± 1.35
R330S	89.60 ± 1.48 (*R*)	85.62 ± 1.38	33.95 ± 0.67 (*R*)	69.67 ± 1.92	64.39 ± 2.05 (*R*)	71.80 ± 1.57
R330C	87.52 ± 1.56 (*R*)	80.50 ± 1.47	33.80 ± 1.41 (*R*)	63.79 ± 1.27	64.35 ± 2.33 (*R*)	70.16 ± 0.86
R330K	86.18 ± 0.84 (*R*)	84.82 ± 2.19	5.45 ± 0.59 (*R*)	63.69 ± 1.51	61.53 ± 1.76 (*R*)	80.64 ± 0.43
R330Q	84.81 ± 1.41 (*R*)	90.74 ± 2.55	10.90 ± 0.84 (*R*)	76.88 ± 0.66	61.17 ± 0.71 (*R*)	85.23 ± 1.44
R330G	92.39 ± 1.66 (*R*)	10.96 ± 0.29	5.07 ± 1.22 (*R*)	8.49 ± 0.15	60.97 ± 1.56 (*R*)	14.82 ± 0.33
R330M	87.88 ± 1.23 (*R*)	92.40 ± 1.77	0.37 ± 0.65 (*R*)	85.18 ± 2.08	58.40 ± 1.08 (*R*)	86.71 ± 1.37
R330Y	87.03 ± 1.12 (*R*)	96.61 ± 2.16	40.92 ± 1.53 (*S*)	88.24 ± 1.89	38.65 ± 1.11 (*R*)	91.56 ± 1.72
R330P ^b^	/	/	/	/	/	/
OYE2y	82.87 ± 0.98 (*R*)	92.20 ± 1.07	32.66 ± 1.77 (*S*)	88.65 ± 1.49	38.13 ± 1.55 (*R*)	89.51 ± 1.68

^a^ X represents any of 20 amino acids. Data present mean values ± SD from three independent experiments. (*E*)-citral contained 98.38% geranial and 1.62% neral, (*Z*)-citral contained 96.84% neral and 3.16% geranial, and (*E*/*Z*)-citral contained 58.45% geranial and 41.55% neral. ^b^ “/” represents no catalytic activity.

**Table 3:** The catalytic performance of OYE2y and its P76X variants ^a^.

Enzyme	(*E*)-Citral		(*Z*)-Citral		(*E*/*Z*)-Citral	
	*e.e*. (%)	Yield (%)	*e.e*. (%)	Yield (%)	*e.e*. (%)	Yield (%)
P76C	85.39 ± 2.70 (*R*)	65.32 ± 0.76	37.50 ± 1.55 (*R*)	38.54 ± 1.61	69.92 ± 2.46 (*R*)	49.65 ± 1.40
P76S	81.23 ± 1.79 (*R*)	81.84 ± 0.62	10.19 ± 0.73 (*S*)	48.15 ± 0.88	62.80 ± 0.97 (*R*)	64.48 ± 0.90
P76M	86.22 ± 0.75 (*R*)	80.39 ± 0.64	4.76 ± 1.50 (*S*)	55.19 ± 0.95	57.60 ± 1.92 (*R*)	61.53 ± 0.89
P76G	85.52 ± 2.55 (*R*)	83.94 ± 0.78	3.63 ± 1.47 (*S*)	57.54 ± 1.84	53.19 ± 0.81 (*R*)	78.04 ± 1.52
P76N	86.45 ± 1.33 (*R*)	85.36 ± 0.49	13.05 ± 0.67 (*S*)	69.39 ± 1.43	49.59 ± 1.55 (*R*)	72.59 ± 0.77
P76A	86.55 ± 0.91 (*R*)	90.57 ± 1.98	21.21 ± 1.95 (*S*)	88.96 ± 0.87	41.88 ± 0.95 (*R*)	90.43 ± 2.22
P76V	87.54 ± 1.55 (*R*)	92.50 ± 2.51	30.15 ± 1.67 (*S*)	82.30 ± 2.07	40.49 ± 1.01 (*R*)	87.37 ± 1.28
P76T	86.12 ± 1.35 (*R*)	93.79 ± 2.26	28.10 ± 1.69 (*S*)	91.24 ± 2.12	37.05 ± 1.13 (*R*)	91.50 ± 0.94
P76L	84.28 ± 1.62 (*R*)	90.95 ± 1.83	38.01 ± 2.27 (*S*)	88.36 ± 1.46	35.05 ± 1.64 (*R*)	88.46 ± 1.32
P76I	86.10 ± 1.03 (*R*)	94.76 ± 1.74	40.23 ± 1.81 (*S*)	89.95 ± 1.33	34.51 ± 1.17 (*R*)	91.88 ± 2.45
P76Y ^b^	/	/	/	/	/	/
P76Q ^b^	/	/	/	/	/	/
P76D ^b^	/	/	/	/	/	/
P76E ^b^	/	/	/	/	/	/
P76R ^b^	/	/	/	/	/	/
P76H ^b^	/	/	/	/	/	/
P76F ^b^	/	/	/	/	/	/
P76W ^b^	/	/	/	/	/	/
P76K ^b^	/	/	/	/	/	/
OYE2y	82.87 ± 0.98 (*R*)	92.20 ± 1.07	32.66 ± 1.77 (*S*)	88.65 ± 1.49	38.13 ± 1.55 (*R*)	89.51 ± 1.68

^a^ X represents one of the other 19 amino acids. Data present mean values ± SD from three independent experiments. (*E*)-citral contained 98.38% geranial and 1.62% neral, (*Z*)-citral contained 96.84% neral and 3.16% geranial, and (*E*/*Z*)-citral contained 58.45% geranial and 41.55% neral. ^b^ “/” represents no catalytic activity.

**Table 4:** The catalytic performance of double substitution variants of OYE2y at sites P76 and R330 ^a^.

Enzyme	(*E*)-Citral		(*Z*)-Citral		(*E*/*Z*)-Citral	
	*e.e*. (%)	Yield (%)	*e.e*. (%)	Yield (%)	*e.e*. (%)	Yield (%)
P76C	85.39 ± 2.70 (*R*)	65.32 ± 0.76	37.50 ± 1.55 (*R*)	38.54 ± 1.61	69.92 ± 2.46 (*R*)	49.65 ± 1.40
P76C/R330H	88.00 ± 2.04 (*R*)	78.09 ± 1.16	76.16 ± 0.88 (*R*)	54.53 ± 0.45	82.32 ± 1.22 (*R*)	57.74 ± 0.84
P76C/R330D	88.77 ± 1.81 (*R*)	80.00 ± 1.52	76.45 ± 1.03 (*R*)	52.23 ± 1.25	81.70 ± 2.18 (*R*)	63.28 ± 1.36
P76C/R330W	89.53 ± 2.23 (*R*)	70.30 ± 1.87	70.32 ± 1.16 (*R*)	56.49 ± 0.68	81.44 ± 1.31 (*R*)	65.31 ± 1.72
P76C/R330C	87.43 ± 1.37 (*R*)	90.10 ± 1.96	49.17 ± 2.44 (*R*)	51.14 ± 0.79	77.37 ± 1.04 (*R*)	66.19 ± 1.42
R330H	88.08 ± 1.39 (*R*)	71.23 ± 0.85	71.92 ± 1.34 (*R*)	64.12 ± 1.33	86.88 ± 1.36 (*R*)	52.83 ± 0.75
P76M/R330H	>99 (*R*)	73.88 ± 2.66	75.01 ± 1.59 (*R*)	13.99 ± 0.23	>99 (*R*)	15.83 ± 0.45
P76G/R330H	>99 (*R*)	64.09 ± 1.32	77.60 ± 2.06 (*R*)	12.64 ± 0.31	>99 (*R*)	11.61 ± 0.38
P76S/R330H	>99 (*R*)	64.74 ± 1.57	74.74 ± 1.84 (*R*)	8.90 ± 0.16	>99 (*R*)	9.12 ± 0.22
OYE2y	82.87 ± 0.98 (*R*)	92.20 ± 1.07	32.66 ± 1.77 (*S*)	88.65 ± 1.49	38.13 ± 1.55 (*R*)	89.51 ± 1.68

^a^ Data present mean values ± SD from three independent experiments. (*E*)-citral contained 98.38% geranial and 1.62% neral, (*Z*)-citral contained 96.84% neral and 3.16% geranial, and (*E*/*Z*)-citral contained 58.45% geranial and 41.55% neral.

## Supplementary Materials

The following are available online at https://www.mdpi.com/1420-3049/24/6/1057/s1, Table S1: The primer information of site saturation mutation of P76 in OYE2y; Table S2: The primer information of site saturation mutation of R330 in Oye2y; Figure S1: SDS-PAGE (12%) analysis of the purified OYE2y R330X variants; Figure S2: SDS-PAGE (12%) analysis of the purified OYE2y P76X variants; Figure S3: Gas chromatography analysis for standards (*S*)-citronellal (22.459 min), (*R*)-citronellal (23.067 min), (*Z*)-citral (29.164 min), and (*E*)-citral (30.398 min); Figure S4: Gas chromatography–mass spectrometry analysis for (*S*)-citronellal (A), (*R*)-citronellal (B), (*Z*)-citral (C), and (*E*)-citral (D) in the asymmetric reduction of (*E*/*Z*)-citral.
