# Supplementary material for: Engineering the Enantioselectivity of Yeast Old Yellow Enzyme OYE2y in Asymmetric Reduction of (E/Z)-Citral to (R)-Citronellal
Source: Molecules. 2019 Mar 18;24(6):1057. doi: 10.3390/molecules24061057 (PMC6470962; doi:10.3390/molecules24061057)
Supplement: Supplementary file 1 [file molecules-24-01057-s001.pdf]

Supporting Information

Engineering the Enantioselectivity of Yeast Old Yellow Enzyme OYE2y in Asymmetric Reduction of (*E*/*Z*)-Citral to (*R*)-Citronellal

Xiangxian Ying [1,*], Shihua Yu [1], Meijuan Huang [1], Ran Wei [1], Shumin Meng [1], Feng Cheng [1], Meilan Yu [2], Meirong Ying [3], Man Zhao [1], Zhao Wang [1]

[1] Key Laboratory of Bioorganic Synthesis of Zhejiang Province, College of Biotechnology and Bioengineering, Zhejiang University of Technology, Hangzhou 310014, China;

[2] College of Life Sciences, Zhejiang Sci-Tech Univeristy, Hangzhou 310018, China;

[3] Grain and Oil Products Quality Inspection Center of Zhejiang Province, Hangzhou 310012, China

* Correspondence: yingxx@zjut.edu.cn; Tel.: +86-571-88320781

Contents

Supplementary tables 2

Table S1. The primer information of site saturation mutation of P76 in OYE2y 2

Table S2 The primer information of site saturation mutation of R330 in Oye2y 4

Supplementary figures 7

Figure S1. SDS-PAGE (12%) analysis of the purified OYE2y R330X variants 7

Figure S2. SDS-PAGE (12%) analysis of the purified OYE2y P76X variants 8

Figure S3. Gas chromatograph analysis for (*S*)-citronellal (22.459 min), (*R*)-citronellal (23.067 min), (*Z*)-citral (29.164 min) and (*E*)-citral (30.398 min) 9

Figure S4. Gas chromatograph-mass spectrometry analysis for (*S*)-citronellal (A), (*R*)-citronellal (B), (*Z*)-citral (C) and (*E*)-citral (D) in the asymmetric reduction of (*E*/*Z*)-citral 10

Supplementary tables

Table S1. The primer information of site saturation mutation of P76 in OYE2y [a]

Primer		Sequence
P76G	F	TACCTTTGGCTCTCCACAATCTGGGGGTT
	R	TGGAGAGCCAAAGGTACCCTCAGTGATAATC
P76A	F	TACCTTTGCCTCTCCACAATCTGGGGGTT
	R	TGGAGAGGCAAAGGTACCCTCAGTGATAATC
P76V	F	TACCTTTGTCTCTCCACAATCTGGGGGTT
	R	TGGAGAGACAAAGGTACCCTCAGTGATAATC
P76L	F	TACCTTTCTCTCTCCACAATCTGGGGGTT
	R	TGGAGAGAGAAAGGTACCCTCAGTGATAATC
P76I	F	GTACCTTTATTTCTCCACAATCTGGGGGT
	R	GTGGAGAAATAAAGGTACCCTCAGTGAT
P76F	F	TACCTTTTTCTCTCCACAATCTGGGGGTT
	R	TGGAGAGAAAAAGGTACCCTCAGTGATAATC
P76Y	F	GTACCTTTTATTCTCCACAATCTGGGGGTT
	R	GTGGAGAATAAAAGGTACCCTCAGTGATAATC
P76W	F	GTACCTTTTGGTCTCCACAATCTGGGGGTT
	R	TGTGGAGACCAAAAGGTACCCTCAGTGAT
P76S	F	TACCTTTTCCTCTCCACAATCTGGGGGT
	R	GTGGAGAGGAAAAGGTACCCTCAGTGAT
P76T	F	GTACCTTTACCTCTCCACAATCTGGGGGTT
	R	GTGGAGAGGTAAAGGTACCCTCAGTGATAATC
P76C	F	GTACCTTTTGTTCTCCACAATCTGGGGGTT
	R	GTGGAGAACAAAAGGTACCCTCAGTGATAATC

P76M	F	GTACCTTTATGTCTCCACAATCTGGGGGTT
	R	GTGGAGACATAAAGGTACCCTCAGTGATAATC
P76N	F	TACCTTTAACTCTCCACAATCTGGGGGTT
	R	GTGGAGAGTTAAAGGTACCCTCAGTGATAATC
P76Q	F	TACCTTTCAGTCTCCACAATCTGGGGGTT
	R	GTGGAGACTGAAAGGTACCCTCAGTGATAATC
P76D	F	GTACCTTTGACTCTCCACAATCTGGGGGTT
	R	GTGGAGAGTCAAAGGTACCCTCAGTGATAATC
P76E	F	TACCTTTGAGTCTCCACAATCTGGGGGTT
	R	GTGGAGACTCAAAGGTACCCTCAGTGATAATC
P76K	F	GTACCTTTAAGTCTCCACAATCTGGGGGTT
	R	GTGGAGACTTAAAGGTACCCTCAGTGATAATC
P76R	F	TACCTTTCGATCTCCACAATCTGGGGGTT
	R	TGTGGAGATCGAAAGGTACCCTCAGTGAT
P76H	F	TACCTTTCACTCTCCACAATCTGGGGGTT
	R	GTGGAGAGTGAAAGGTACCCTCAGTGATAATC

[a] The code to introduce the substitution was underlined.

Table S2. The primer information of site saturation mutation of R330 in Oye2y [a]

Primer		Sequence
R330F	F	GCTCTGTTCCCAGAAGTTGTCAGAGAAG
	R	TTCTGGGAACAGAGCAAAGTTACCAGCT
R330W	F	GCTCTGTGGCCAGAAGTTGTCAGAGAAG
	R	TTCTGGCCACAGAGCAAAGTTACCAGCT
R330P	F	GCTCTGCCCCCAGAAGTTGTCAGAGAAG
	R	TTCTGGGGGCAGAGCAAAGTTACCAGCT
R330Y	F	GCTCTGTACCCAGAAGTTGTCAGAGAAG
	R	TTCTGGGTACAGAGCAAAGTTACCAGCT
R330M	F	GCTCTGATGCCAGAAGTTGTCAGAGAAGAGGT
	R	TTCTGGCATCAGAGCAAAGTTACCAGCTC
R330G	F	GCTCTGGGCCCAGAAGTTGTCAGAGAAG
	R	TTCTGGGCCCAGAGCAAAGTTACCAGCT
R330A	F	GCTCTGGCCCCAGAAGTTGTCAGAGAAG
	R	TTCTGGGGCCAGAGCAAAGTTACCAGCT
R330V	F	GCTCTGGTCCCAGAAGTTGTCAGAGAAG
	R	TTCTGGGACCAGAGCAAAGTTACCAGCT
R330L	F	GCTCTGCTCCCAGAAGTTGTCAGAGAAG
	R	TTCTGGGAGCAGAGCAAAGTTACCAGCT
R330I	F	GCTCTGATCCCAGAAGTTGTCAGAGAAG
	R	TTCTGGGATCAGAGCAAAGTTACCAGCT
R330S	F	GCTCTGAGCCCAGAAGTTGTCAGAGAAG
	R	TTCTGGGCTCAGAGCAAAGTTACCAGCT

R330T	F	GCTCTGACCCCAGAAGTTGTCAGAGAAG
	R	TTCTGGGGTCAGAGCAAAGTTACCAGCT
R330N	F	GCTCTGAACCCAGAAGTTGTCAGAGAAG
	R	TTCTGGGTTCAGAGCAAAGTTACCAGCT
R330Q	F	GCTCTGCAGCCAGAAGTTGTCAGAGAAG
	R	TTCTGGCTGCAGAGCAAAGTTACCAGCT
R330D	F	GCTCTGGACCCAGAAGTTGTCAGAGAAG
	R	TTCTGGGTCCAGAGCAAAGTTACCAGCT
R330E	F	GCTCTGGAACCAGAAGTTGTCAGAGAAG
	R	TTCTGGTTCCAGAGCAAAGTTACCAGCT
R330K	F	GCTCTGAAGCCAGAAGTTGTCAGAGAAG
	R	TTCTGGCTTCAGAGCAAAGTTACCAGCT
R330C	F	GCTCTGTGCCCAGAAGTTGTCAGAGAAG
	R	CTTCTGGGCACAGAGCAAAGTTACCAGCT

[a] The code to introduce the substitution was underlined.

Supplementary figures

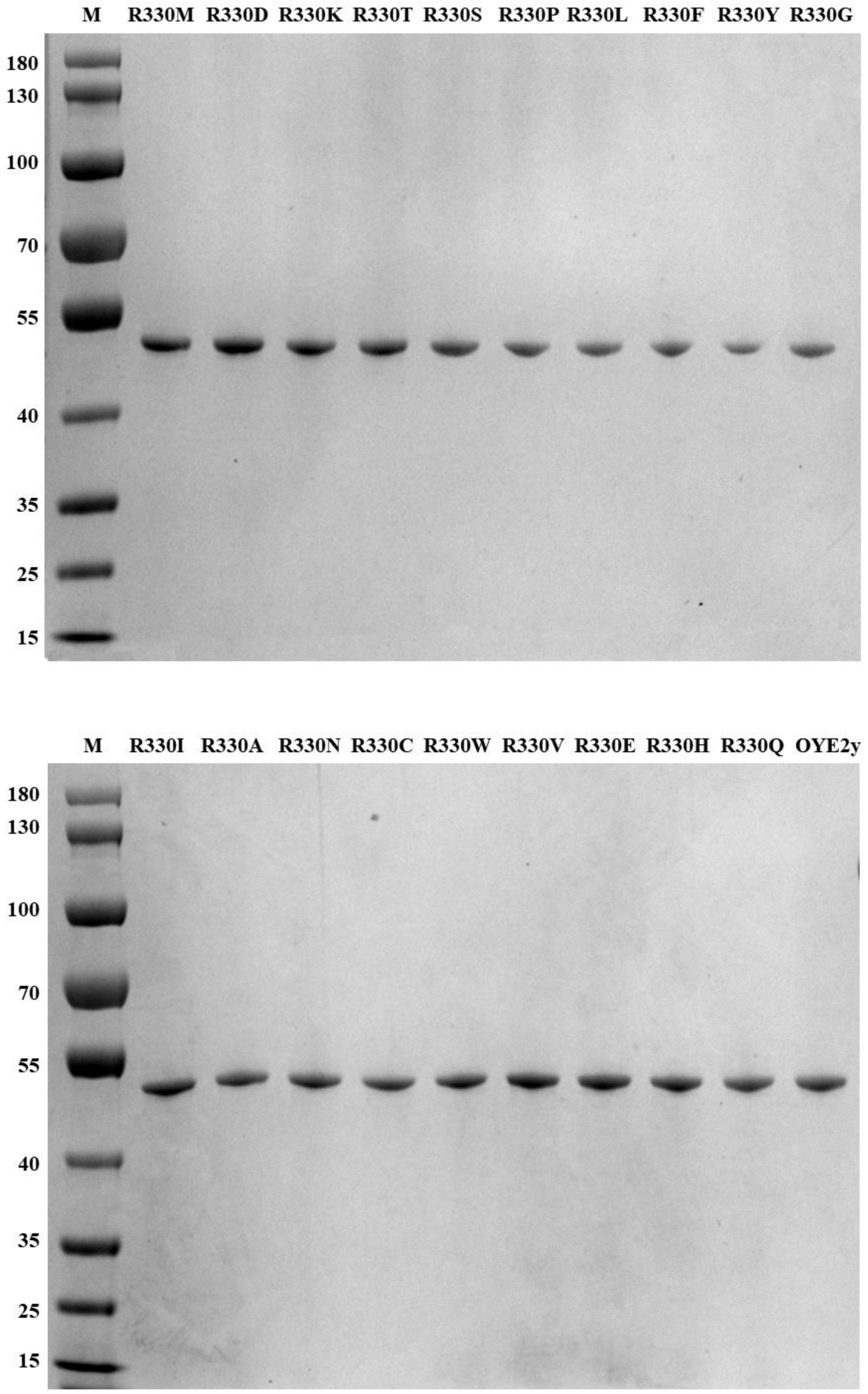

Figure S1. SDS-PAGE (12%) analysis of the purified OYE2y R330X variants. Lane M, standard molecular mass proteins. Other lanes are named according to the substitution for OYE2y. The proteins were visualized by staining with Coomassie brilliant blue R-250.

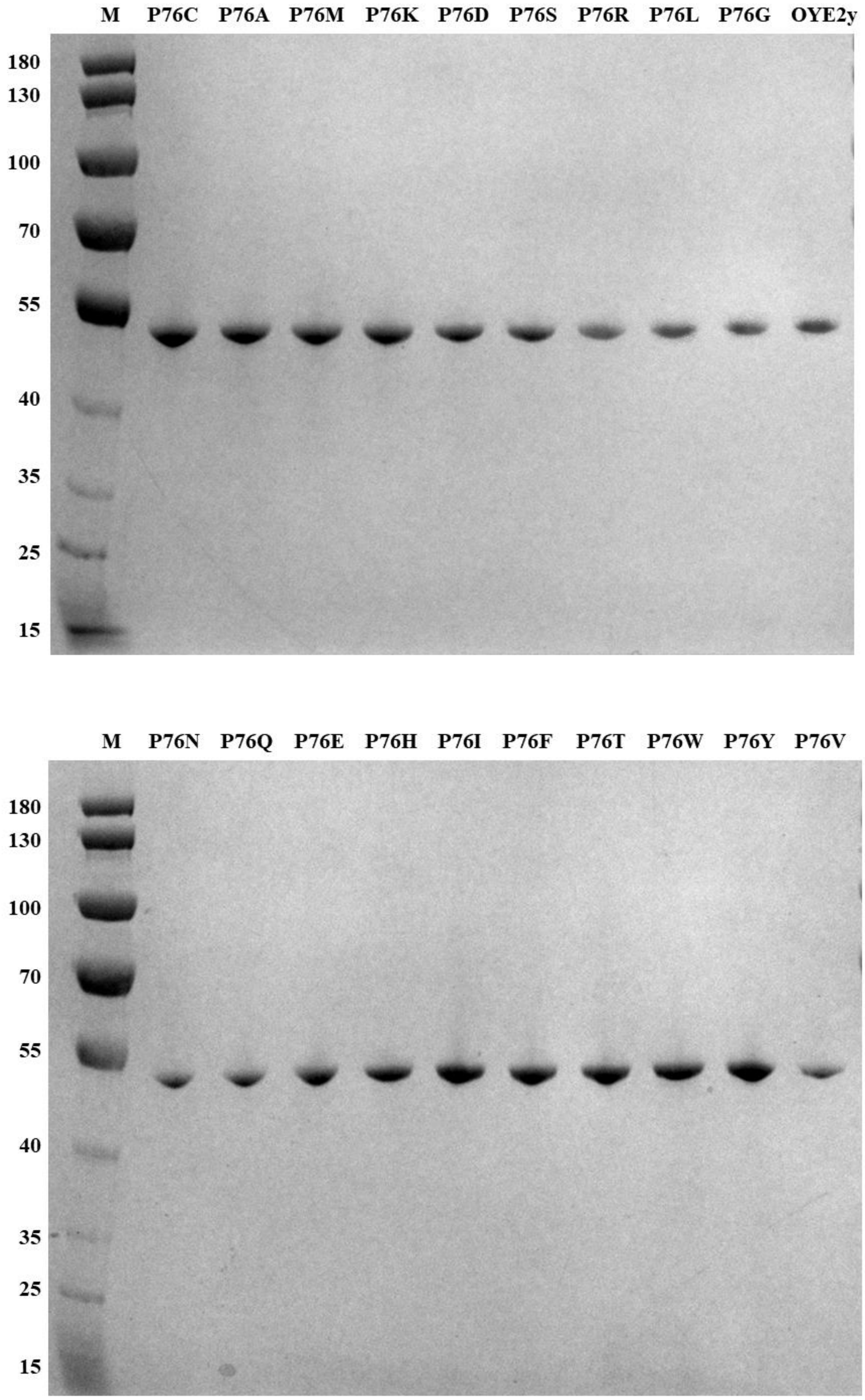

Figure S2. SDS-PAGE (12%) analysis of the purified OYE2y P76X variants. Lane M, standard molecular mass proteins. Other lanes are named according to the substitution for OYE2y. The proteins were visualized by staining with Coomassie brilliant blue R-250.

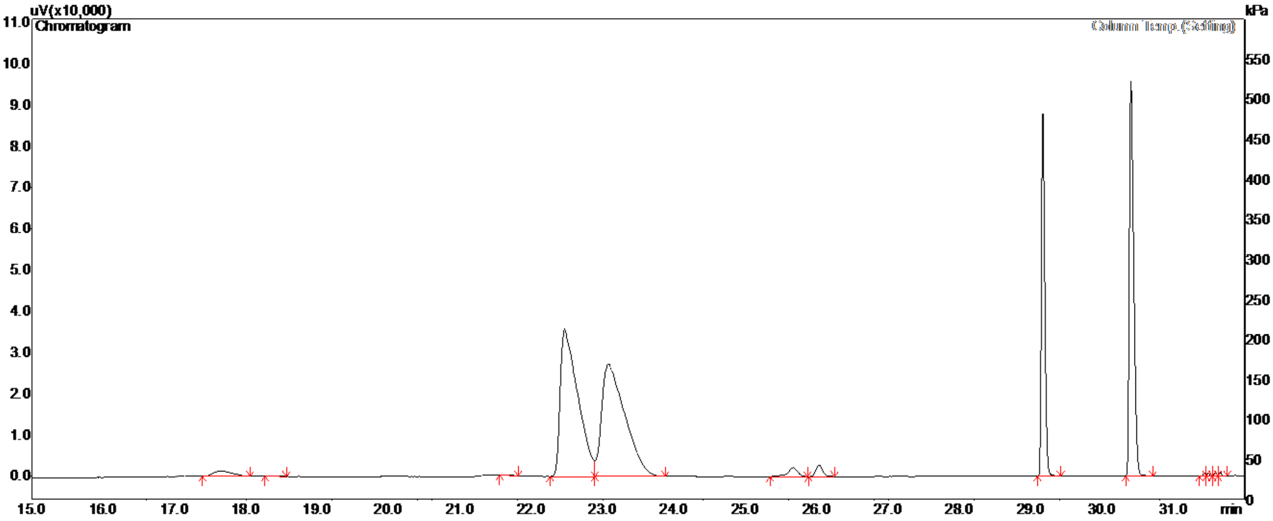

Figure S3. Gas chromatograph analysis for standards (*S*)-citronellal (22.459 min), (*R*)-citronellal (23.067 min), (*Z*)-citral (29.164 min) and (*E*)-citral (30.398 min).

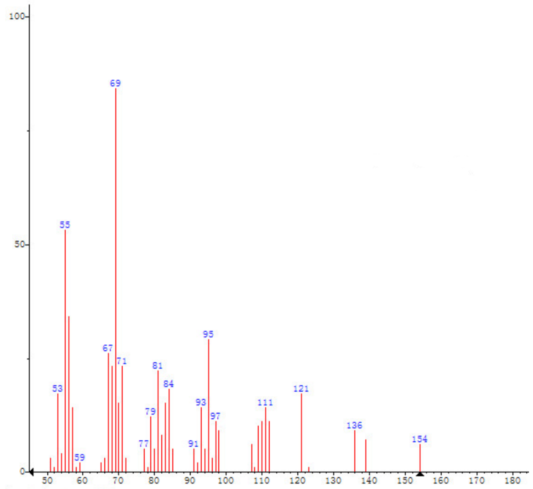

A

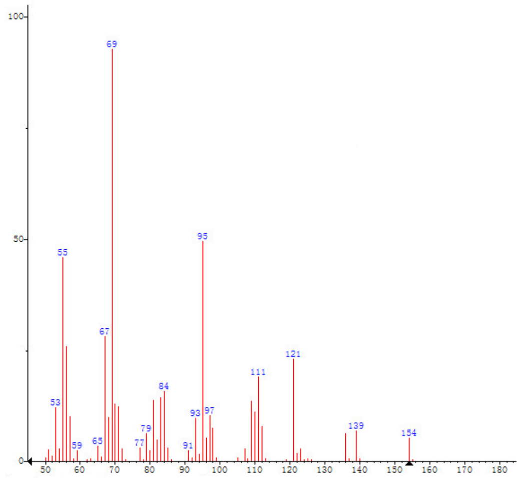

B

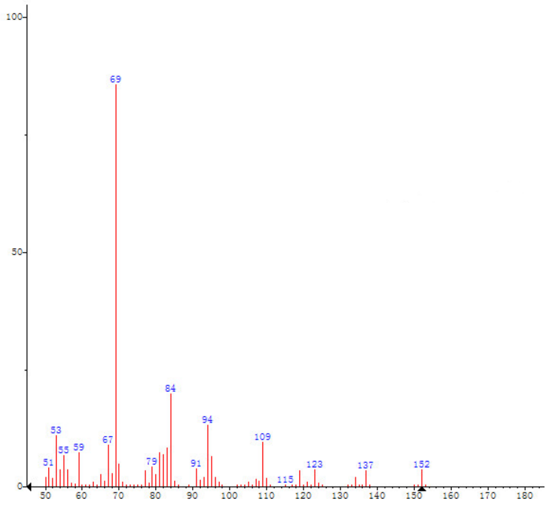

C

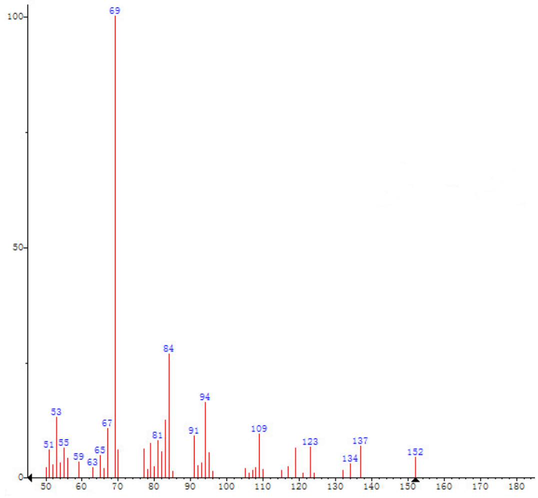

D

Figure S4. Gas chromatograph-mass spectrometry analysis for (*S*)-citronellal (A), (*R*)-citronellal (B), (*Z*)-citral (C) and (*E*)-citral (D) in the asymmetric reduction of (*E*/*Z*)-citral.
